# Teachers’ language use in United Kingdom Chinese community schools: Implications for heritage-language education

**DOI:** 10.3389/fpsyg.2022.899428

**Published:** 2022-08-11

**Authors:** Androula Yiakoumetti

**Affiliations:** School of Education, Oxford Brookes University, Oxford, United Kingdom

**Keywords:** L1 use, Mandarin Chinese, crosslinguistic practices, teacher-training programmes, heritage-language education, complementary education

## Abstract

This study deals with teachers’ language use as it is manifested in community-based heritage-language classes. Specifically, it focuses on the functions of students’ dominant variety (L1, English) when harnessed by teachers for the purposes of teaching their ethnic language (L2, Mandarin Chinese). Empirical investigation was conducted at two Chinese community schools in the United Kingdom and data demonstrate that students’ L1 was utilised naturally and systematically by teachers to facilitate students’ L2 learning. Various L1 facilitative functions were identified and these generally accord well with functions recorded in other studies. In addition, this study underlines the potentially unique characteristics of community-based heritage-language education: while the target variety of students (Mandarin Chinese) is routinely the native variety of teachers, teachers struggle to explain new linguistic information in Mandarin because of students’ low proficiency while they concurrently struggle with at least some elements of students’ native or dominant variety (English). Teachers explained that the fact that students’ dominant language of English is a global language makes their use of it all the more likely. Teachers demonstrated a strong tendency to feel ill-prepared for their language teaching role. There is thus a clear need for heritage-language teachers to receive training that is sociolinguistically informed. This training should emphasise the potential utility of exploiting students’ full linguistic repertoires by drawing in particular on the crosslinguistic similarities and differences between the varieties of which students are cognisant. Teacher-training programmes that promote such awareness may well hold the key to better heritage-language education which will continue to have a crucial role in maintaining and developing minority community languages.

## Introduction

Over the last fifty years, the role of students’ native or dominant varieties when learning and teaching additional varieties has regularly attracted a great deal of research attention. Numerous theoretical propositions suggested by various scholars including Atkinson (1987, 1993), Martin-Jones (1995), Cook (2001), Ferguson (2003, 2009) and Cummins (2011) have been supported by empirical research carried out in diverse linguistic, geographical, and educational settings (Canagarajah, 1995; Eldridge, 1996; Moore, 2002; Yiakoumetti and Mina, 2013a; Cahyani et al., 2018; Parba, 2018; Lucas and Yiakoumetti, 2019; Cancino and Diaz, 2020; Ataş and Sağın-Şimşek, 2021). Both theoretical discussions as well as practical investigations suggest that it is immensely beneficial to utilise students’ dominant varieties when teaching and learning additional varieties. It should be briefly observed that deployment of students’ dominant language in learning and teaching additional languages is not always regarded as beneficial and that dominant languages are thus often neglected or even banned. For a review of this debate, see Littlewood and Yu (2011) and Ma (2019).

Because a primary focus of this special issue is to inform the research field regarding the manner in which students’ dominant variety (L1) may positively influence their additional languages (L2), special consideration is given to studies that deal with L1 in L2 teaching, codeswitching, and translanguaging. In fact, crosslinguistic pedagogical practices (Cummins, 2019) that reflect any natural languaging that takes place in the daily lives of linguistically-diverse students are judged to be appropriate (and, indeed, necessary) for a meaningful and authentic learning experience.

It is necessary at this point to briefly address a recent paradigm shift in educational sociolinguistic research. Educational sociolinguistics in the 21st century is questioning the diglossic compartmentalisation which was at the heart of educational practices in the 20^th^ century (García, 2009). While target linguistic varieties in education were once kept separate from students’ home or dominant varieties (because such home varieties were viewed as interfering forces), recent teaching methodologies have evolved to embrace broad repertoire building (Cummins, 2021). For the purposes of this study, research that is conducted within the frameworks of both codeswitching and translanguaging is examined. Codeswitching is an established topic of research that has attracted the interest of scholars for a number of decades and, whilst research into tranlanguaging only emerged (and burgeoned) within the last 10 years (Lewis et al., 2012), both have very significant research corpora associated with them. Although pedagogical codeswitching and translanguaging each have their faithful followers who point out the main differences between codeswitching and translanguaging (see Li, 2018), scholars do not shy away from embracing both codeswitching and translanguaging in their research frameworks (see Park, 2013; Faltis, 2019; Ataş and Sağın-Şimşek, 2021; Sahan and Rose, 2021). Research that takes into account linguistic resources from the entire linguistic repertoires of multilingual speakers represents the core background of this study. Furthermore, this study seeks to echo Cenoz and Gorter’s (2020, 2021) pedagogical translanguaging principles which consider it fundamentally problematic to fail to utilise learners’ prior knowledge to support emerging discourses. This study also endorses Cummins’ (2021) theoretical views on crosslinguistic educational practices that explicitly challenge the exclusion of some of the linguistic resources of multilingual learners whilst calling for more explicit attention to the development of multilingual language awareness by focussing on crosslinguistic similarities and differences amongst students’ varieties.

Previous research in language classrooms has mainly focussed on these three aspects:

1. instances of shuttling between teachers’ and learners’ first language and the target second/foreign language (Kharma and Hajjaj, 1989; Duff and Polio, 1990; Franklin, 1990; Polio and Duff, 1994; Rolin-Ianziti and Brownlie, 2002; Yiakoumetti and Mina, 2013b; Narayan and Kuar, 2022),
2. instances of shuttling between teachers’ and learners’ first dialect and the target second dialect (Battisti et al., 2011; Malcolm and Truscott, 2012; Ayiomamitou and Yiakoumetti, 2017), and
3. establishing links between crosslinguistic educational practices and student learning and performance (Yiakoumetti, 2006, 2007; Then and Ting, 2011; Lucas and Yiakoumetti, 2019).

As current research rationally favours utilisation of students’ entire linguistic repertoires rather than exclusive teaching in the target variety, this empirical study aimed to first identify instances of such utilisation before suggesting practical recommendations for a more effective, inclusive and empowering education. The vantage point of this study is heritage-language education (also known as ‘ethnic’ or ‘complementary’ education) as implemented in Chinese community schools in the United Kingdom. This study comes as a response to the calls including those by Macaro (2001) and Turnbull and Arnett (2002) for further research into the role of teachers’ language practices, by Creese and Martin (2006) for further investigation of complementary-school education, and by Francis et al. (2009) for further studies into Chinese complementary education. Community schools serve as ideal sites for the exploration of the language practices of teachers who very often do not share the same dominant language as that of their students (Ganassin and Holmes, 2020). In addition, and importantly, community schools in the United Kingdom (and, indeed, in other English-speaking settings) are unique in that the dominant language of students is usually the all-powerful English. It would be reasonable to argue that students’ dominant language in such settings may thus have a distinctive role within heritage-language education.

It is worth noting that a great deal of research on the utilisation of students’ entire linguistic repertoires is associated with the English-as-a-foreign-language (EFL) field and focuses on teachers’ and learners’ utilisation of their mother tongues to facilitate the learning of English as a foreign language. As mentioned earlier, this study deals with the different but burgeoning domain of teaching Chinese as an ethnic language in the United Kingdom. Over the last two decades, Chinese language teaching in the United Kingdom, and especially Chinese language teaching in complementary schools, has significantly advanced (Wang and Higgins, 2008; Francis et al., 2009; Mau et al., 2009; Zhang and Li, 2010) and it is likely to expand further as a result of the ongoing rise of the Chinese economy and the rise in the numbers of both Chinese immigrants and UK-born Chinese. Another reason that Chinese-language education is expected to expand is that Chinese has recently been identified as one of the languages that is important for the UK’s future: looking at a variety of economic, geopolitical, cultural and educational indicators, Tinsley and Board (2013) score Chinese in fourth place (after Spanish, Arabic and French) as being vital for the UK’s prosperity, security and influence in the world over the next 20 years.

## Teachers’ language practices in linguistically-diverse settings

The role of teachers’ language practices in students’ performance has received considerable research attention. This research has pointed to a direct link between target-language achievement and language choice which has consequently led some researchers to argue for maximal teacher use of the target language (e.g., Turnbull, 2001). However, as van Lier (1995) and Cook (2001) observe, the suggestion to maximise use of the target language in the classroom has been misinterpreted by many educators who took it to mean that they should avoid or, at best, restrict the use of students’ first or dominant languages. Ma (2019) additionally explains that one of the reasons that the role of students’ native language has been neglected in EFL teaching is because the classroom is sometimes the only domain for target-language exposure in EFL contexts.

Indeed, in many linguistically-diverse classrooms around the world, teachers’ utilisation of students’ native varieties (L1) is viewed negatively and with suspicion by teachers themselves (with a similar view being reflected in formal language policies). For instance, teachers interviewed by Mitchell (1988) about their use of L1 believed that they committed professional misconduct. Similarly, teachers interviewed by Probyn (2009) believed that L1 use is to be avoided. In Flanders (the Dutch-speaking part of Belgium), van de Craen and Humblet (1989) reported that teachers felt ashamed when told that they use nonstandard native varieties in the classroom and these teachers concluded that they could not speak in the way they felt they should. It is obvious that many teachers feel guilty or ashamed when they employ a non-target variety in the class (Carless, 2008). At the policy level, many language policies around the world prescribe the use of the target language (L2 or D2) alone and view codeswitching and translanguaging as undesirable and harmful. This view is undoubtedly due to policies being influenced by pervasive and persistent monolingual ideologies. Phillipson (1992) astutely gives the label of ‘the monolingual fallacy’ to the idea that language is best taught monolingually.

Exclusive use of the target language is unrealistic and this is especially true when teachers deal with foreign languages. Despite policies requiring exclusive use of the target language policies and teachers’ best intentions, utilising students’ dominant varieties is reasonable and expected. The very occurrence of this utilisation highlights the fact that it can serve a number of interactional and pedagogical functions that enhance students’ learning. Cook (2001) convincingly argues that teachers ought to utilise students’ L1 if they wish to create a more authentic learning experience for students.

Researchers have recently reported cases in which the L1 was employed purposefully and successfully by teachers. Raschka et al. (2009) explain that EFL teachers in Taiwan switch between Mandarin and English for reasons including socialising, topic switching, classroom management and metalinguistic functioning. Tien’s (2009) study, also in Taiwan, supports these findings and adds to the repertoire of reasons for codeswitching: teachers commonly switch from English to Mandarin to unlock meanings from monolingual English textbooks and to promote harmony in the classrooms. Inbar-Lourie (2010) summarised the purposes of EFL teachers’ use of students’ L1 in Hebrew- and Arabic-medium schools into instructional, managerial and affective. Interestingly, Cleghorn (1992) explains that teachers in Kenya are more successful in conveying important ideas when they do not adhere to the English-only language policy but, instead, switch to one of the three indigenous languages of Kenya (Kiswahili, Kikuyu and Luo). Along similar lines, Ahmad and Jusoff (2009) demonstrate that teachers and students regularly switch to Malay, the common language amongst the multilingual students in English classes in Malaysia. This language use serves a number of functions and enhances students’ learning experience. Drawing on the language choices of teachers in South Africa, Adendorff (1993) highlights that switches from English to Zulu have important academic and social functions: they guide academic activity as well as the interpretation of social information. Students’ opinions about their teachers’ use of their L1 also highlight the many beneficial functions associated with the use of students’ dominant languages. For studies that investigate L1 functions in teachers’ practices as recalled by students, see Littlewood and Yu (2011) (for students from Hong Kong and mainland China learning English) and Bhooth et al. (2014) (for students from Yemen learning English).

The potential arising from the use of students’ native varieties in the classroom is immense. As demonstrated above, a plethora of studies on teachers’ language use evidences the fact that such utilisation serves as a tool for students that is conducive to learning (e.g., Macaro, 2001; Kraemer, 2006; García, 2009; Hobbs et al., 2010; García et al., 2012; Wang, 2019; Ataş and Sağın-Şimşek, 2021, in addition to those mentioned above). It is thus reasonable to expect the classroom to resemble life outside its boundaries where navigating between various varieties is viewed as a natural and valuable resource for bidialectal, bilingual, emerging bilingual, and multilingual speakers (Jacobson, 1990).

## Heritage-language education

This study focuses on the provision of Chinese as a heritage (or ethnic) language. Specifically, this study explores United Kingdom Chinese community education. Community schools, also known as ‘complementary’ or ‘supplementary’ schools, predominantly serve immigrant and ethnic communities in the multilingual United Kingdom. (For a historical overview of the broad types of complementary schooling in the United Kingdom, see Li, 2006). Complementary schooling is conducted outside the state sector and is primarily designed by and for immigrant communities in an effort to maintain their linguistic and cultural heritage (Yiakoumetti, 2015). For instance, Greek, Polish, and Chinese community schools serving British-born generations of these communities can be found throughout the United Kingdom. In the last 30 years, there has been enormous voluntary commitment to the teaching of community languages by minority communities themselves (Kempadoo and Abdelrazak, 2001) and it was estimated that, in 2009 in the United Kingdom, there were over 3,000 complementary schools which offered language instruction in over 80 languages (CILT, 2009; Maylor et al., 2010).

Although complementary schools in the United Kingdom have existed for a number of decades, with the exception of some notable work (e.g., Li, 1993; Martin et al., 2003; Language and Education, 2006; Francis et al., 2009; Li and Wu, 2009; Mau et al., 2009; Ganassin, 2020), the field is considered to be under-researched and under-theorised (Hall et al., 2002). Alarmingly, there has been little collaboration between complementary schools and mainstream education (Anderson and Macleroy, 2015). Beyond the United Kingdom, research on heritage/community language education is expanding in the United States, Canada and Australia (Baldauf, 2005; Elder, 2005; Mercurio and Scarino, 2005; Tucker, 2005; Curdt-Christiansen, 2006; Brinton et al., 2008; Leeman, 2015).

This study comes at a time when the Chinese community schools in the United Kingdom are faced with exciting opportunities as well as practical challenges (Ganassin and Holmes, 2020). The opportunities stem from the increasing attention that the teaching and learning of Chinese has recently attracted. The challenges mainly relate to a lack of suitable teaching staff and teaching materials. It is reasonable to concur with Kagan (2005) who argues that, for pedagogical purposes, ethnic-language speakers should not be considered as either native or foreign speakers of the ethnic language because they are a part of a unique population. This population must negotiate between majority and minority languages and identities and thus requires its own curriculum and materials.

This study also comes at a time when the Chinese language is enjoying heightened attention in the broader British community: it is present in many community schools, secondary schools, specialist language colleges and universities, and even in some primary schools (For studies that address the expansion of teaching Chinese worldwide, see Lo Bianco, 2007; Wang, 2019; Bao et al., 2020). This study is timely because it draws attention to schools that promote alternative discourses at times when, as May (2012a) observes, a rapid and significant retrenchment of multilingualism and multiculturalism within education can be observed. In Europe as well as in the US, bilingual educational programmes face significant devaluation as minority groups are increasingly urged to strive towards dominant cultural and linguistic mores (Crawford, 2007; Modood, 2007; May, 2012a,b).

Indeed, although Britain is one of the most multilingual settings in the world, there is unfortunately a disregard for true multilingualism and the official language policy is unquestionably anachronistic. Discourses around multilingualism tend to promote monolingualism or support bilingual educational provision which favours languages with perceived prestige status (Lanvers, 2011; Anderson and Macleroy, 2015; Yiakoumetti, 2015). Disappointingly, the study of languages in British schools has declined in recent years (Board and Tinsley, 2014). Community language schools are thus viewed as immensely important for appreciating, safeguarding and developing community identities and languages. Community schools promote linguistic diversity through times in which the monolingual ideology still reigns supreme (Creese and Martin, 2006).

## Project

### Aims of the study

The primary aim of this study was to investigate the manner in which teachers employed students’ dominant language, English, in their teaching of Chinese as a heritage language. Specifically, this study set out to identify the functions of students’ dominant language. This aim was inspired by the call for more research into teachers’ actual language use as opposed to teachers’ and students’ perceptions of such use. A secondary aim of this study was to identify teachers’ views on their language choices within the heritage-language educational context. This aim presented itself based on research that demonstrates that heritage-language learners cannot be assigned neatly into traditional language-learner categories because they are neither native nor foreign-language speakers. Finally, where appropriate, this study aimed to use its findings to make suggestions for improving heritage-language education. In summary, these are the three key research questions:

1. What are the various functions of teachers’ use of students’ dominant language?
2. What are teachers’ views on their language use within heritage-language education?
3. How can heritage-language teachers be better equipped to optimise their language teaching?

### Setting of the study

This study was conducted in two Chinese community schools: one in Cambridgeshire and one in Oxfordshire (United Kingdom). To preserve anonymity, the schools are not named here. The participating schools offer two-hour Mandarin and Cantonese language classes of various levels on weekends. The majority of the student population are British-born Chinese students whose dominant language is English and who attend these schools in an effort to acquire, maintain or improve their proficiency in Chinese and to enhance their knowledge of the Chinese culture. Students range from four to 17 years of age and they are assigned to different classes based on their language proficiency rather than their age. This method of assignment is common in United Kingdom Chinese community schools (Li, 1993). Most teachers are Chinese-speaking community members or graduate students at the local universities.

### Participants

The main participants of the project were four teachers whose language practices were investigated. In compliance with the preferences of the heads of each school, teachers who taught Beginners’ Mandarin Chinese were chosen. Students were thus at the lower end of heritage-language ability. While the majority of them heard Chinese at home, they only rarely employed the language themselves. This study focused on the four teachers’ utilisation of their students’ dominant language (English) during the teaching of the target heritage language (Mandarin/Putonghua). The teachers originated from mainland China with Mandarin being their first language and English a foreign language. All had been teaching at the complementary schools for a minimum of a year prior to the commencement of this study and all volunteered to participate in this study. Two of the four teachers had formal teaching qualifications. Ethical procedures ensured teachers’ anonymity. Importantly, teachers had the option of withdrawing from the project at any time.

### Procedure

The project was carried out over a term (i.e., a three-month period) and employed a qualitative research design. The main data-collection tools were (1) video recordings of teachers’ classroom language use and (2) teacher interviews. Four sessions were recorded for each teacher which amounted to approximately 32 h of video-recorded data. Analysis of these data focused on teachers’ utilisation of students’ L1 dominant language, English. This utilisation was firstly categorised. Once the L1 functions were categorised, two independent critical auditors who were fluent in both Mandarin and English reviewed these for accuracy. Subsequently, each teacher was interviewed individually. At the interview, the teachers were shown video recordings of instances in which they utilised students’ L1 and were asked to comment on their language use, whether they were aware of their practices, to elaborate on their attitudes towards their linguistic behaviour, and to discuss their personal views about the role of L1 when teaching an ethnic language.

## Findings and discussion

### L1 functions

Unexpectedly, analysis of teachers’ language use revealed that they *primarily* used students’ dominant L1 (English) during lessons. L1 use was employed purposefully and had a number of functions. Table 1 lists the twenty-eight functions that were identified with an example and an English translation for each function. The twenty-eight functions are presented in order of decreasing frequency in Table 1. Some of the most prominent functions are discussed. It must be noted at this point that, at times, it was less than straightforward to identify teachers’ exact purposeful use of students’ L1 simply because the natural languaging of the classroom was primarily conducted in students’ L1.

**Table 1:** Teachers’ utilisation of students’ L1 (English) in the L2 (Mandarin) community-based heritage-language classroom.

L1 function	Example	English translation
1. Explanation of new vocabulary	星期一 、星期二、 星期三、 星期四 、星期五、 星期六。In Chinese, we use 星期 and numbers for each day of the week. Monday 星期一, Tuesday星期二, Wednesday 星期三, Thursday星期四, Friday星期五、 Saturday星期六。	Monday, Tuesday, Wednesday, Thursday, Friday, Saturday. In Chinese, we use ‘week’ and numbers for each day of the week. Monday, ‘Monday’, Tuesday, ‘Tuesday’, Wednesday, ‘Wednesday’, Thursday, ‘Thursday’, Friday, ‘Friday’, Saturday, ‘Saturday’.
2. Extension	这是什么?青椒。This is 青椒. 这是青椒。	What is this? Pepper. This is a pepper. This is a pepper.
3. End marker	你在整理吗? Finished?	Are you tiding up? Finished?
4. Checking understanding	这是铅笔。对吗? Is this correct?	This is a pencil. Is this correct? Is this correct?
5. Rapport	Are you OK? 我们来贴ok绷。	Are you OK? Let us put on the plaster.
6. Repetition	今天是婷婷的生日, it is Ting Ting’s birthday today. It is her birthday. 我们來唱生日快乐歌。	Today is Ting Ting’s birthday, it is Ting Ting’s birthday today. It is her birthday. Let us sing a birthday song.
7. Support beyond academic matters	我们来吃点心。慢慢吃Slow。喝水。Drink your water.	Let us have some snacks. Eat slowly. Slow. Drink water. Drink your water.
8. Drawing attention (and Explanation)	我们来练习颜色!绿色、黄色、红色。Look! There is a hot air balloon in the sky. Hot air balloon热气球, red hot air balloon, 红色的热气球。	Let us practice the colours! Green, yellow, red. Look! There is a hot air balloon in the sky. Hot air balloon, hot air balloon, red hot air balloon, red hot air balloon.
9. Explanation of grammatical rules	白色的衣服, white top. 粉红色的手套, pink gloves. 蓝色的车子, blue car. Do you understand the grammar rule? The adjective is before the noun, like in English.	White top, white top. Pink gloves, pink gloves. Blue car, blue car. Do you understand the grammar rule? The adjective is before the noun, like in English.
10. Provision of metalinguistic knowledge	三颗橘子, three oranges. 二颗苹果, two apples. In Chinese, you do not need ‘s’ to show many. One orange, two orange, one apple, two apple.	Three oranges, three oranges. Two apples, two apples. In Chinese, you do not need ‘s’ to show many. One orange, two orange, one apple, two apple.
11. Motivation to practice more	Teacher: 大拇指、食指、中指、无名指、小拇指 Student: 大拇指、食指、中指、无名指、小拇指 Teacher: Very good, 好棒, again, 再一次 Student: 大拇指、食指、中指、无名指、小拇指	Teacher: Thumb, index finger, middle finger, ring finger, little finger Student: Thumb, index finger, middle finger, ring finger, little finger Teacher: Very good, very good, again, again. Student: Thumb, index finger, middle finger, ring finger, little finger
12. Correction	Teacher: 你赢了! Student: 你赢了! Teacher: No! it is我赢了, I won.	Teacher: You won! Student: You won! Teacher: No! It is I won, I won.
13. Description of target culture	Moon Festival, 中秋节, we normally have a BBQ on that day.	Moon Festival, Moon Festival, we normally a have BBQ on that day.
14. Disciplining	Sit down, 坐下。Be quiet, 安静。我们来说丑小鸭的故事。	Sit down, sit down. Be quiet, be quiet. Let us have the story of Ugly Duckling.
15. Emphasising	我有个惊喜要给你。Surprise, 惊喜!我给每个人一颗粽子。	I have a surprise for you. Surprise, surprise! I will give everyone a Zongzi.
16. Replacing unknown words for teachers	我们来玩滑梯?秋千? Zip line?	Let us play on the slide? Swing? Zip line?
17. Loanwords	有什么在零食盒?草莓、蓝莓、cheese, 起司。	What is in the snackbox? Strawberries, blueberries, cheese, cheese.
18. Beginning marker	Right! 我们翻开第20页。	Right! Let us turn to page number 20.
19. Substitution	谁想要看TV?	Who wants to watch TV?
20. Praising	棒!Good! 很棒!Very good!你最棒, you are the best!	Good! Good! Very good! Very good! You are the best, you are the best!
21. Shifting behaviour	Stop crying, 不要哭, 停! Stop! Let us call your mum.	Stop crying, stop crying, Stop! Stop! Let us call your mum.
22. Joking	我忘记带国语课本, 我好笨, I’m stupid.	I forgot to bring the Chinese textbook, I’m stupid, I’m stupid.
23. Self-correction	你的零食盒有草莓、蓝莓、芹菜、oh sorry, cucumber.	There are strawberries, blueberries, celery, oh sorry, cucumber.
24. Explanation of pronunciation	眼睛、鼻子、头。 头just like “toe”	Eyes, nose, head. Head just like “toe”
25. Linkage with known linguistic and cultural topics	今天, 我们来念三只小猪的故事。三只小猪, Three Little Pigs.	Today, we are reading the story of the three little pigs. Three Little Pigs, Three Little Pigs.
26. Explanation of cultural aspects of language	“好” means a woman and a man together.	“Good” means a woman and a man together.
27. Elicitation of students’ answers	我们来数1到10。 1、2、3、4、5、6、7、8、9、10。Now we count in 10s. 十、二十、三十、四十、五十、六十。 Do you understand the rule? How do we count in 10s? 十、二十、三十、四十、五十、六十 … continue (all students count together) 七十、八十、九十。	Let us count 1 to 10. 1, 2, 3, 4, 5, 6, 7, 8, 9, 10. Now we count in 10s. 10, 20, 30…40, 50, 60. Do you understand the rule? How do we count in 10s? 10, 20, 30, 40, 50, 60… continue (all students count together) 70, 80, 90.
28. Classroom management	谁知道答案?Raise your hand.	Who knows the answer? Raise your hand.

All teachers systematically reverted to students’ dominant language, English, to introduce and explain new vocabulary and grammatical structures. This L1 function of explanation was the most common function. Teachers’ language choice was based on their belief that first-language provision facilitated the acquisition of new linguistic knowledge and they explained that they arrived at this belief through their own foreign-language learning experiences. Teachers additionally stated that, because students were only just starting to learn Mandarin, there was not much scope for teachers to use the target language meaningfully. They further explained that resorting to English was the only option in many instances because a number of students have Cantonese-speaking parents and thus do not get any exposure to Mandarin at home.

Students’ L1 was employed by all teachers for extension and provision of metalinguistic knowledge. During interviews, teachers explained that these L1 functions serve authentic needs in communication. Extensions help elicit additional responses from students and provision of metalinguistic knowledge ensures students’ understanding. Teachers viewed such L1 functions as an indispensable natural tool.

End markers produced in students’ L1 were very common. However, during the interviews, teachers expressed surprise at the frequency of their end markers. They admitted to feelings of guilt for employing this type of L1 function but rationalised that it successfully served their aim of extracting students’ immediate responses and, consequently, saving valuable lesson time.

In their efforts to build rapport and assist students with personal matters (i.e., support beyond academic matters), teachers purposefully employed students’ first language. They elaborated that their choices were driven by their beliefs that students associated the second language with teaching and the first language with more familiar, casual and comfortable settings.

The L1 functions presented above are congruent with educational and linguistic research. Pan and Pan (2010) categorised existing research on teachers’ L1 use into three groups. The first group focuses on access and includes functions such as conveying meaning and explaining grammar. The second group focuses on L1 use for classroom management and includes functions such as organising tasks, disciplining, and praising students. The third group focuses on L1 use for interpersonal relations and includes functions such as telling jokes. As a general remark, the teachers of this study utilised students’ L1 for all three overarching purposes with the most common occurrence relating to the explanation of grammar rules and unknown lexicon.

A recent empirical study carried out by Ataş and Sağın-Şimşek (2021) classified teachers’ utilisation of students’ L1 (Turkish) in EFL classrooms into two broad groups: educational and discourse functions. The former group includes functions such as extending, explaining and emphasising while the latter group includes functions such as self-repair, inviting participation and personalising for emotions. Again, the teachers of the current study utilised these overarching groups of functions in a purposeful manner. This study also accords well with Inbar-Lourie’s (2010) study on teachers’ utilisation of students’ L1 (Hebrew or Arabic) in the EFL classrooms. The main L1 functions recorded in her study—instructional (e.g., grammatical and lexical explanations), managerial (e.g., classroom management and feedback provision) and affective (e.g., rapport and care)—were also employed by the teachers of this study. Furthermore, the findings of the current study coincide with those of other studies. For instance, they support Kang’s (2008) study of a Korean EFL teacher which demonstrated that the teacher used the L1 for functions such as explanation of grammar, organisation of tasks, disciplining of students, and implementation of tests. Similarly, there is alignment with Franklin’s (1990) study that reported that many teachers preferred the L1 for disciplining and Edstrom’s (2006) study that reported that L1 helps establish rapport and solidarity with students. In addition, the current study accords well with Harbord’s (1992) recommendation that teachers employ the L1 to tell jokes such that student anxiety is reduced. Finally, teachers’ actions are certainly in accordance with Atkinson’s (1987) proposal that teachers should explain grammatical rules in the students’ mother tongues such that these rules are reinforced.

Although it does not pertain directly to teachers’ language use, it is warranted to address an issue that was raised by all of the participants. During the interviews, teachers were asked to describe whether they use any specific strategies that incorporate students’ L1 to facilitate the learning and teaching of their L2. All teachers referred to drawing attention to salient characteristics of Mandarin that differ from English. They explained that this strategy of juxtaposing students’ L1 and L2 is beneficial because it provides the metalinguistic awareness that so often facilitates language acquisition and production. The crosslinguistic metalinguistic information presented in Table 2 was identified by all teachers as being crucial.

**Table 2:** Teachers’ comments on essential metalinguistic information that compares students’ L1 (English) and L2 (Mandarin).

L1–L2 comparison	Example (English)	Example (Mandarin)
Lack of plural markings in Mandarin	tree – trees	树–树
Verbs are not conjugated in Mandarin	I eat – she eats	我吃 – 她吃
Adjectives precede nouns in both Mandarin and English	red dress	红色的裙子
Subject-Verb-Object sentence structure in both Mandarin and English	I have a doll	我有一个洋娃娃

### Teachers’ views on their language use and the uniqueness of heritage-language education

Undoubtedly, some of the most striking data from this study relate to the unique setting of community-based heritage-language education. A selection of the teachers’ comments that clearly demonstrate this uniqueness is presented in Boxes 1, 2. The first set of comments includes those that are associated with the use of students’ dominant L1 (English) and target language (Mandarin) and the second set is associated with comments on other factors that affect teaching in complementary schools.

BOX 1 **Teachers’ comments on why they use students’ L1 (English) in L2 (Mandarin) community-based heritage-language classrooms.**

1. When I first came here, I thought that I would be using Chinese but most children do not speak well. Their parents send them to us but some children do not want to be here. They do not want to speak Chinese. They do not do their homework.
2. Sometimes I use more English than I would like.
3. If we use more Chinese, the children will not understand.
4. Some parents speak to their children in Mandarin and the children are happy to use some Mandarin. Other children refuse to speak in Mandarin. Some parents are Cantonese speakers but they want their children to learn Mandarin … it’s difficult. Our class is too big with children of various abilities. We need to divide the class but we do not have teachers.
5. Teaching here can be challenging. We’ve recently asked students to buy their own textbooks but a parent complained because the textbook is about Chinese as a foreign language. The mother did not want her child to be called a foreign speaker.
6. I wished that I was given guidelines about how much English I’m expected to use in the lessons.
7. Some of my colleagues who came from China recently were shocked to see that students here do not speak Chinese. I’m used to it. I have a daughter who comes to this school and her Chinese has improved. But she goes to school every day and she only speaks English.
8. My English is not fluent. Sometimes it is difficult to explain new concepts in English.

BOX 2 **Teachers’ comments on factors that influence teaching in Mandarin community-based heritage-language classrooms.**

9. It is hard to find teachers who are willing to work here because of the money. The headmistress has to work hard to find money to pay the teachers because of funding cuts. That’s why we cannot give textbooks to students anymore. We used to. Now we ask them to buy them.
10. In my class, I have a girl who is young and does not know how to read in English yet but her Chinese is better than her other classmates. What can I do? I still teach pinyin but she does not know how to read.
11. We have families from all walks of life. Some send their children to private schools. Others work in restaurant kitchens. … We need money so we had to raise the fees.
12. The parents are motivated. They want their children to speak Chinese and to learn about Chinese culture.
13. When parents help students with their homework, students feel more confident in class.
14. I have a boy who comes to class with his older sister. She speaks Chinese fluently because she was born in China. He was born here. I do not mind that she comes along. She helps him.
16. Short training before the year starts would be helpful. I know that there is not money for it but it would help us understand what we are dealing with.

All teachers reported that they systematically and consciously harnessed students’ L1. Their individual comments had a common theme: awareness that the L1was regularly employed for the smooth running of every lesson. At the same time, when shown recordings of their language use, they all reported in a number of instances that they were unaware of the fact that they used English (L1). The fact that teachers were not always aware of their language choices is not surprising. Utilising one’s entire linguistic repertoire is so natural for linguistically-diverse speakers that it is routinely performed subconsciously. Other researchers have also alluded to the fact that teachers are not always conscious of their codeswitching practices (Adendorff, 1993; Yiakoumetti and Mina, 2013a). However, a sense of surprise and even guilt and regret was conveyed through teachers’ discourse as they believed that they should have used students’ target language in certain occasions. It is unfortunate that teachers expressed such concerns because using students’ strong languages in a second-language class is associated with positive outcomes (Auerbach, 1993).

As it is evident from teachers’ first set of comments, teachers struggled to determine how much English vs. Mandarin to use during lessons. Despite teachers’ initial expectations, it rapidly became apparent that using the target language, Mandarin, primarily was not conducive to learning. They reported that they found it difficult to explain new linguistic information in Mandarin due to students’ low proficiency in target-language tasks. By the same token, all teachers explained that, at times, they struggled similarly to convey meaning in students’ dominant language, English, because English was not native for them. Ironically, all teachers were of the opinion that teaching Mandarin as a heritage language in the United Kingdom was distinctive because teachers nevertheless have access to students’ dominant language (English) even though this access may be relatively superficial. Teachers elaborated that, because everyone has at least some knowledge of English, opting for the convenience of using this shared language is unavoidable.

Although teachers held mostly positive views towards their use of students’ strong language to facilitate student learning, they concurrently felt uncertain about their linguistic behaviour. This uncertainty led them to believe that they were somewhat ill-equipped for teaching Mandarin as an ethnic language. They explained that, during the time that they had been teaching at the complementary schools, their views regarding teaching Mandarin to ethnically Chinese British-born children changed. Concerns relating to parents’ views of their children’s Chinese learning were identified: a teacher felt challenged by the fact that a parent did not wish for her child to be labelled as a speaker of Chinese-as-a-foreign-language. Such challenges undoubtedly affect teachers and this is unfortunate because the consequent constraints upon pedagogical practices.

Teachers expressed a desire for training in issues relevant to ethnic-language education. This finding highlights the need for more attention to be paid to training teachers who intend to teach in Chinese community schools. Being native speakers of the language as well as qualified teachers from mainland China does not seem to be sufficient in the heritage-language context: the teachers within this study lacked confidence and felt unprepared for this unique educational context. If teachers are more sociolinguistically informed about the language habits of students who attend complementary schools, they may well be in a better position to make and justify language choices that are suited to their specific environment.

It should be emphasised that, even though this study focused on teachers’ language use, minority children’s ethnic-language education in community schools is influenced by a number of other factors. It is beyond the scope of this article to discuss those other factors in detail but it is important to at least briefly mention some of the most crucial ones. It is for this reason that the second set of teachers’ comments was included in Box 2. It is evident that financial issues, students’ varying degrees of Chinese competence, teaching materials, and sociolinguistic background are all factors that affect heritage-language education in complementary schools. These findings align with those of other researchers working within the United Kingdom heritage-language landscape (e.g., Wang, 2014).

There is no doubt that the teachers consider the role of family to be at the forefront of successfully maintaining and developing heritage-language competence. Indeed, many theoretically- and empirically-based studies have demonstrated that grandparents, older relatives such as aunts and uncles, and siblings all help to provide an environment conducive to ethnic-language use (Pauwels, 2005; Gregory et al., 2007; Kenner et al., 2007). Li (1993) argued that, to make community language education more effective, parents should be encouraged to get involved with members outside of their immediate community.

Another suggestion which relates to heritage-language education (but which was not raised by the teachers) comes from Borland (2005) who argues that technology may play a key role in the learning of ethnic languages for diasporic communities. The author explains that virtual learning alone will not be sufficient but the power of technology should be harnessed in the learning of minority ethnic-language education.

This study provides evidence that teachers should ideally undergo training in how to teach ethnic minority languages. This study thus endorses its teachers’ views that it is not sufficient in and of itself for native Chinese teachers to serve as educators in such schools. These teachers require training in the linguistic habits and the language attitudes of the students who attend community schools. Li et al. (1992) and Li (1994) explain that British-born Chinese children speak English most of the time, unlike their parents and grandparents. Many of these children feel pressure to attend Chinese-language classes and some consider community language education of little use (Wong, 1992). By gaining knowledge of the sociolinguistic realities of the educational context, teachers will be in a better position to successfully motivate and to subsequently educate their students. To better equip teachers, training programmes thus ought to take into account language habits, language attitudes, teacher limitations, and teaching materials as well as financial and practical constraints.

## Conclusion

Using one’s entire linguistic repertoire is a natural phenomenon that occurs in linguistically-diverse settings around the world (including multilingual, bilingual, emergent bilingual, multidialectal, bidialectal and diglossic settings). It forms part of the daily lives of people and it serves a number of functions. In fact, everyone shuttles regularly between varieties and we all possess linguistically-diverse competence: monolinguals shuttle between codes, registers and discourses while multilinguals also shuttle between their various languages (Canagarajah, 2011). Despite the fact that codeswitching and translanguaging are largely regarded as both common in speech and a valuable resource outside the classroom boundaries, they are still considered inappropriate in many classroom contexts (Li and Martin, 2009). They are thus often either proscribed or accepted but unsanctioned in teacher training. Educational sociolinguists, however, have repeatedly argued that utilising students’ dominant languages is a valuable pedagogical and communicative resource which should be better harnessed by teachers in the classroom (e.g., Yiakoumetti, 2011).

This study focused on community-based heritage-language education and, specifically, on teachers’ language practices. This is due to the fact that teachers’ role is viewed as being immensely significant as they potentially hold the key for transmitting both linguistic and cultural knowledge relating to children’s ethnic identity. This study provides evidence that supports the potential utility and benefits of deliberate use of students’ L1. Teachers employed students’ dominant language as a successful pedagogical strategy. L1 use was natural and served pedagogical, interpersonal and communicative purposes. Harnessing students’ L1 is thus viewed as a pedagogical tool that could help maintain and develop diversity. By utilising students’ dominant languages, their heritage second languages have more chance of surviving. It is argued that teachers’ utilisation of all of the students’ linguistic resources should be celebrated in the classroom and treated as an undeniably useful tool.

Every sociolinguistic and educational setting is unique and this study has highlighted some of the distinctive characteristics of (1) heritage-language education and (2) Chinese as a heritage language in the United Kingdom.

1. Heritage-language education cannot be reduced or equated to foreign-language education. Students’ parents and even students who do not speak their heritage language nevertheless have a special affinity to their ethnic language and the culture that accompanies it. This strong bond with the language and culture of their ancestors (even in cases in which it does not translate into practice) ought to be harnessed for language acquisition and development. Teachers must thus be equipped so that they are in a position to marry the two cultures of ethnic minority students: only when the heritage culture and the wider community’s culture are brought more closely together will students become more receptive towards their two identities. (For research that investigates the ways in which community-school teachers and students understand Chinese culture, see Ganassin, 2019). Appropriate sociocultural and sociolinguistic classroom discussions would assist students in understanding that their two identities need not operate in conflict. When such attitudinal shift occurs, preservation of heritage languages pursues. This responsibility rests largely on the shoulders of teachers.
2. Chinese as a heritage language in the United Kingdom is unique due to the undeniable role of English (the wider community’s and students’ dominant language) as an international and all-powerful language. Students very often do not see a reason for learning other languages because of the unprecedented power of English. Indeed, research attests to the fact that success in raising children to be bilingual remains the exception in the United Kingdom (and the United States; Fillmore, 2000). This eventuality is due to the high status of English. As community-based heritage-language education serves as one of the limited opportunities available for ethnically-Chinese children to learn their familial language, its role is indispensable. For this reason, the teachers who are the main transmitters of knowledge in this system should be appropriately trained and empowered to realise their invaluable purpose.

The findings in this study are derived from observations of the teaching of Chinese as a minority ethnic language in just two British community schools. The implications of these findings for Chinese teaching in the United Kingdom are nevertheless clear: lack of proper teacher training leads to teachers’ lack of confidence. The teachers who participated in this study expressed concerns over their suitability to teach Chinese as an ethic language. It is paramount that teachers of community-based ethnic languages be made aware of the unique sociolinguistic and educational settings in which they teach. Improvement of teachers’ understanding and appreciation of the functions and symbolic representations of the linguistic varieties of the ethnic community would empower them such that they become increasingly confident and effective.

The work reported here leads to a significant question: how can teachers in linguistically-diverse settings (e.g., D2, L2, L3) be equipped to optimise their language teaching? The answer is clear: sociolinguistically-informed teacher-training programmes that

1. celebrate language diversity,
2. focus on the detection of salient features through cross-linguistic instruction and
3. utilise the benefits to be had from awareness of L1-L2 similarities and differences and comprehend that these have the potential to allow teachers and, in turn, their students to appreciate and make use of all the linguistic varieties available to them. Even though this study was implemented within a community-based heritage-language educational context, such suggestions may well have a much more universal applicability. It would not be unreasonable to call for all teachers of linguistically-diverse students to be exposed to appropriate sociolinguisitically-informed training.

Community languages are beneficial for both individuals and society. As Gibson (2007) argues, community languages potentially have the ability to change the United Kingdom for the better as knowledge of languages has direct implications for intercultural relations, academia, and business. Competencies surrounding linguistic diversity facilitate global work, study and travel in addition to trade and investment. Beyond the natural desire to do so, there are thus distinct incentives to maintain and develop these critical competencies. Complementary language schools are already offering much-needed education that rejects unhelpful ideologies of monolingual majority languages. The critical role of complementary schools deserves increased recognition and support.

## Data availability statement

The original contributions presented in the study are included in the article/supplementary material, further inquiries can be directed to the corresponding author.

## Ethics statement

Ethical review and approval was not required for the study on human participants in accordance with the local legislation and institutional requirements. The patients/participants provided their written informed consent to participate in this study.

## Author contributions

The author confirms being the sole contributor of this work and has approved it for publication.

## Conflict of interest

The author declares that the research was conducted in the absence of any commercial or financial relationships that could be construed as a potential conflict of interest.

## Publisher’s note

All claims expressed in this article are solely those of the authors and do not necessarily represent those of their affiliated organizations, or those of the publisher, the editors and the reviewers. Any product that may be evaluated in this article, or claim that may be made by its manufacturer, is not guaranteed or endorsed by the publisher.
